# Intermediate-term impact on corneal endothelial cells and efficacy of Preserflo MicroShunt implantation in patients with open-angle glaucoma – a prospective study over two years

**DOI:** 10.1007/s00417-024-06508-8

**Published:** 2024-05-21

**Authors:** Caroline J. Gassel, Daniel A. Wenzel, Emil Nasyrov, Torsten Strasser, Bogomil Voykov

**Affiliations:** 1 University Eye Hospital Tuebingen, Centre for Ophthalmology, Elfriede-Aulhorn-Str. 7, Tübingen, 72076 Germany; 2 Institute for Ophthalmic Research, Centre for Ophthalmology, Tuebingen, Germany

**Keywords:** Preserflo MicroShunt, Corneal endothelial cells, Open-angle glaucoma, Glaucoma surgery, Microinvasive bleb surgery

## Abstract

**Introduction:**

Preserflo MicroShunt is a novel microinvasive bleb forming device for the treatment of primary open-angle glaucoma. The intermediate- and long-term success and the impact of this procedure on corneal endothelial cell density remain to be investigated.

**Methods:**

In this prospective observational study, 62 eyes of 55 glaucoma patients (mean age ± SD: 67.0 ± 15.0 years) receiving a Preserflo MicroShunt were included. Corneal endothelial cell density, intraocular pressure and best corrected visual acuity were assessed preoperatively and at 3, 6, 9, 12, 18 and 24 months postoperatively. Success rates, bleb revision rates and complications were analysed. Complete success was defined as an intraocular pressure reduction of ≥ 20% and achieving a target pressure of ≤ 18, ≤ 15 or ≤ 12 mmHg without antiglaucoma medication. Qualified success indicated that the criteria were reached with or without medication.

**Results:**

Corneal endothelial cells showed no significant decline over 24 months (*p* > 0.05). Intraocular pressure showed a substantial reduction postoperatively (*p* < 0.001), decreasing from 29.6 ± 8,3 mmHg to 13.0 ± 4.3 mmHg after 24 months (*p* < 0.001). Complete and qualified success with a target pressure ≤ 15 mmHg was achieved in 52.9% and 54.6% of cases after 24 months, respectively. Best corrected visual acuity did not change after 24 months.

**Conclusion:**

Preserflo MicroShunt had no negative side effects on corneal endothelial cells and showed favourable success rates after 2 years in patients with open-angle glaucoma.


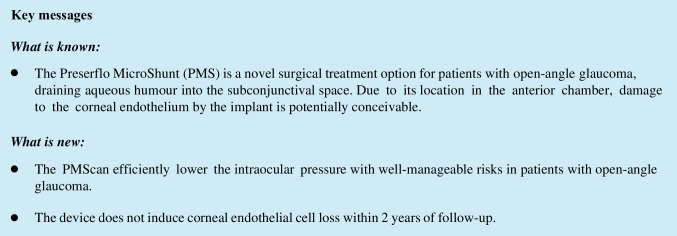


## Purpose

Intraocular pressure (IOP) is the only modifiable risk factor of glaucoma progression [1]. When medical treatment with IOP-lowering eye drops is either insufficient or not possible due to adherence issues, surgery is necessary to prevent progressive visual field defects. Microinvasive bleb surgery with subconjunctival devices such as the XEN Gel Stent (AbbVie Inc., North Chicago, Illinois, USA) and the Preserflo MicroShunt (PMS) (Santen Pharmaceutical Co., Osaka, Japan) have recently gained popularity due to a better risk profile than trabeculectomy [2]. However, there are concerns regarding the impacts of the various stents on the corneal endothelium, due to the experience with the supraciliary CyPass Micro-Stent. This device was withdrawn from the market due to increased corneal endothelial cell (CEC) loss 5 years after implantation [3]. As a result, clinicians are showing considerable interest in determining the effectiveness and safety profile of stent-related filtering surgery, especially regarding CECs.

The PMS obtained its CE marking in 2012 [4] and received FDA approval in July 2020 [5]. The stent is made of a highly biocompatible and bioinert synthetic material called poly(styrene-block-isobutylene-block-styrene), abbreviated as SIBS. Measuring 8.5 mm in length, it has an outer diameter of 350 µm and an inner lumen diameter of 70 µm [6]. The device is implanted ab externo, creating a subconjunctival filtering bleb. Similar to other filtration surgeries, mitomycin C (MMC) is used intraoperatively to modulate the postoperative wound healing. The aim of our study was to investigate the intermediate-term efficacy and safety of the PMS in patients with open-angle glaucoma over a follow-up period of 2 years, with a focus on its potential effects on CECs.

## Methods

This was a prospective, observational, nonrandomized study of patients who underwent PMS implantation. The study was conducted at a single tertiary centre. All surgeries were performed by a single, experienced surgeon (BV). None of the first 50 patients receiving the PMS at our centre were included in this study in order to reduce bias due to learning curve effects.

Inclusion criteria comprised the diagnosis of primary open-angle glaucoma, pigmentary glaucoma or pseudoexfoliative glaucoma, with above-target IOP despite the maximum tolerated IOP-lowering medication, and a minimum follow-up period of 12 months. Both pseudophakic and phakic patients were included, and all procedures were performed as solo operations without additional phacoemulsification.

Exclusion criteria were the diagnosis of narrow-angle glaucoma or normal tension glaucoma and a history of corneal diseases or corneal transplantation.

The study adhered to the principles of the Declaration of Helsinki of 1964. Prior to surgery, all patients provided informed consent, along with additional written consent for study participation. Ethical approval was granted by the local institutional ethics committee of the University of Tübingen (project number: 037/2021BO2).

### Outcome measures

The primary outcome of the study was the mean change in IOP after 2 years. Secondary outcomes included change in CEC density, number of IOP-lowering medications, complete success rate, needling rate and complication rate after 2 years. Complete success was defined as an IOP reduction ≥ 20% from baseline and achieving a respective target IOP of ≤ 18 mmHg, ≤ 15 mmHg or ≤ 12 mmHg without IOP-lowering medications. If this IOP target was reached with or without IOP-lowering medication, the success was labelled as qualified. Hypotony was defined as IOP < 6 mmHg. Any further glaucoma surgery, except needling and incisional bleb revision, and the loss of light perception were defined as failure. Each active agent in combinations of IOP-lowering medications was counted as an individual treatment.

For an additional statistical analysis of success and bleb revision rates, we divided the study cohort into two groups: patients with previous glaucoma surgery (“previous surgery”, including prior trabeculectomy, XEN gel stent, canaloplasty, deep sclerectomy and trabeculotomy) and patients without previous surgery (“no previous surgery”). Patients who received only selective laser trabeculoplasty or diode laser cyclophotocoagulation prior to PMS implantation were also categorized as “no previous surgery”.

### Surgical technique

The surgical procedure was performed under parabulbar anaesthesia. A traction suture was placed through the superior peripheral cornea. The conjunctiva was incised using two radial incisions, followed by blunt dissection of the subconjunctival space. Two round sponges soaked with 0.2 mg/mL MMC were placed under the conjunctiva for 3 min. Then, the sponges were removed, and the area was rinsed with balanced salt solution. A 1 mm-wide tunnel incision was made 3.5 mm from the limbus, and the PMS was introduced into the anterior chamber. After confirming the presence of fistulation through the PMS, the conjunctiva was closed with 10–0 nylon sutures. Dexamethasone and mezlocillin were injected subconjunctivally at the end of the procedure.

### Pre- and postoperative management

IOP-lowering medication was stopped 2 weeks prior to surgery. Oral acetazolamide (250 mg) was administered twice daily until the night before surgery. Starting 1 week before surgery, unpreserved dexamethasone eye drops were instilled three times daily. No medication was administered on the day of surgery.

Postoperatively, moxifloxacin eye drops were initiated four times daily for 1 week beginning on the first postoperative day. Additionally, unpreserved dexamethasone eye drops were gradually tapered over a period of 7 weeks, starting with five drops per day on the first postoperative day.

Patients were examined on the first 2 postoperative days. Further follow-up visits were scheduled after 2 weeks and after 1, 3, 6, 12, 18 and 24 months.

In cases of inadequate IOP control or upon detecting clinical signs of bleb scarring, either a needling procedure or an incisional bleb revision was attempted. Needling was performed under topical anaesthesia using a needle to gently disrupt fibrotic tissue covering the PMS. A total of 25 µL of 0.2 mg/mL MMC (5 µg) was injected into the filtering bleb. Incisional bleb surgery was performed when the PMS was not visible subconjunctivally at the slit lamp. In these cases, the conjunctiva was incised, and the fibrotic tissue surrounding the PMS was dissected, and MMC was applied via soaked sponges. Moxifloxacin eye drops were administered four times daily for 3 days, and unpreserved dexamethasone eye drops were gradually tapered over a 5-week period after both needling and incisional bleb surgery.

### Statistical analysis

The statistical analysis was performed using JMP 16.0 statistical software (SAS Institute Inc., Cary, NC, USA). Data were presented as frequency (percentage), mean (standard deviation [SD]), mean (standard error of the mean [SEM]), mean (95% confidence interval [95% CI]) or median (range), as appropriate. A linear mixed-effects model was used to analyse the effect of PMS implantation on best corrected visual acuity (BCVA), IOP and endothelial cell density (ECD). Dunnett’s test was used to compare pre- and postoperative BCVA, IOP and ECD. Changes in the number of IOP-lowering medications were assessed with the Wilcoxon signed-rank test. Kaplan–Meier analysis was used to calculate rates of complete and qualified success. A *p*-value of < 0.05 was considered to reflect a significant difference.

## Results

### Characteristics of the study population

Sixty-two eyes of 55 patients were included in the study. Patients’ baseline characteristics are shown in Table 1. The mean (± SD) age was 67 (± 15) years, and 54.8% of the studied eyes were from female patients. Thirty-eight eyes (61.3%) were pseudophakic and 24 eyes (38.7%) were phakic at the time of PMS implantation. The majority of eyes (60.3%) underwent no previous glaucoma surgery or laser treatment.

**Table 1:** Baseline characteristics of the study population

Demographic and ocular characteristics of the study population		
Characteristic	*N*	%
Number of eyes (right/left)	62 (36/26)	100 (58.1/41.9)
Age in years, mean ± SD	67.0 ± 15.0	
Gender (female/male)	34/28	54.8/45.2
Lens status (phakic/pseudophakic)	24/38	38.7/61.3
Preoperative hypotensive drug classes, mean ± SD	3.2 ± 0.9	
Prior glaucoma surgery or laser treatment		
Procedure	*N*	%
Selective laser trabeculoplasty	6	9.7
Diode laser cyclophotocoagulation	9	14.5
XEN Gel Stent	7	11.3
Trabeculectomy	6	9.7
Canaloplasty	1	1.6
Deep sclerectomy	2	3.2
Trabeculotomy	1	1.6

### Development of IOP control, BCVA and medication

The mean (± SD) IOP decreased significantly from 29.6 (± 8.3) mmHg at baseline to 14.3 (± 5.3) mmHg after 12 months and to 13.0 (± 4.3) mmHg after 24 months (*p* < 0.001).

**Fig. 1:**
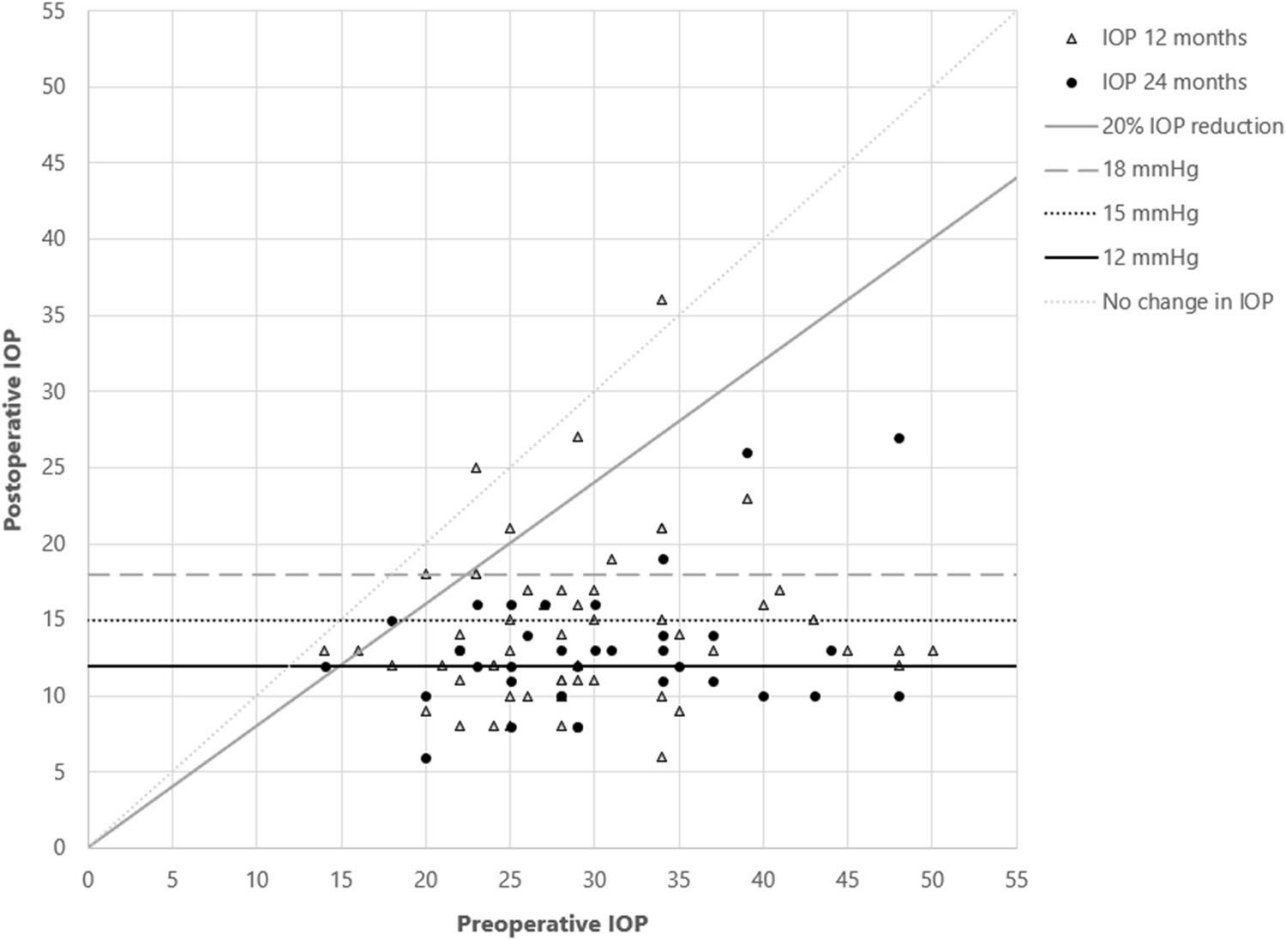
Scatterplot of IOP values preoperatively vs 12 months and 24 months after PMS implantation, respectively. Cut-off IOP levels of ≤ 12 mmHg, ≤ 15 mmHg and ≤ 18 mmHg are depicted. The grey dotted line marks no change in IOP pre- to postoperatively. The grey solid line indicates a 20% reduction in IOP

We observed average IOP reductions of 48.5% and 54.8% after 12 and 24 months, respectively. A comparison of pre- versus postoperative IOP values after 12 and 24 months is shown in Fig. 1.

**Fig. 2:**
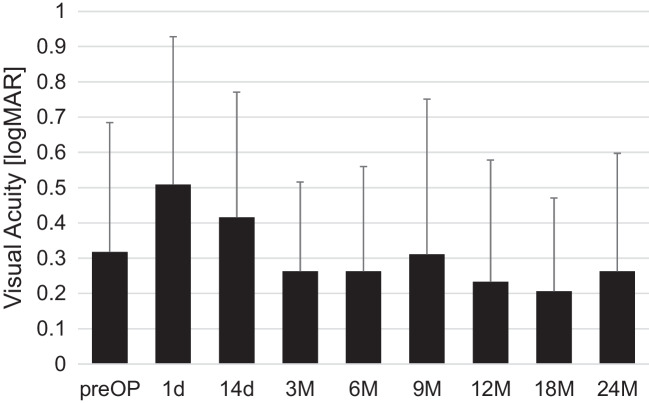
Mean and SD BCVA at all study follow-up time points

BCVA declined significantly from 0.3 (± 0.3) logMAR at baseline to 0.5 (± 0.4) logMAR at day 1 and to 0.4 (± 0.4) logMAR at day 14 postoperatively (*p* < 0.001) but did not differ significantly from preoperative BCVA at any additional follow-up time points (*p* > 0.05). Twelve months after PMS implantation, BCVA was 0.2 (± 0.3) logMAR (*p* > 0.05), and it did not change significantly after 24 months (0.3 ± 0.3 logMAR; *p* > 0.05). The mean and SD BCVA at each follow-up time point are shown in Fig. 2.

The mean (± SD) number of IOP-lowering medications was reduced significantly from 3.2 (± 0.9) at baseline to 0.2 (± 0.7) after 12 months and to 0.2 (± 0.8) after 24 months (*p* < 0.001).

### Endothelial cell density

**Fig. 3:**
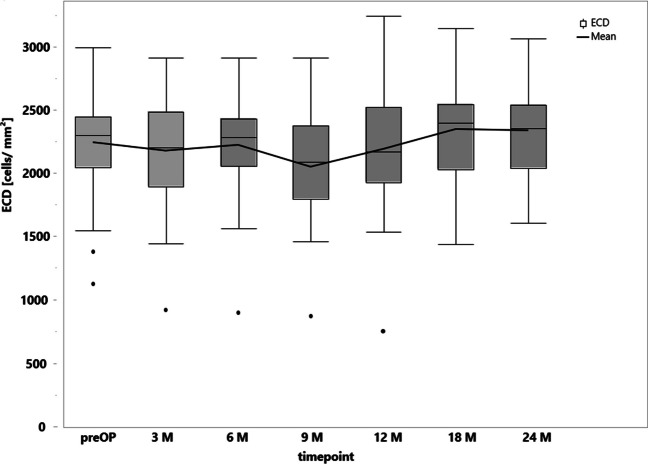
Boxplots showing median, first quartile, third quartile, minimum and maximum ECD at baseline and 3, 6, 9, 12, 18 and 24 months after PMS implantation. The mean values are shown graphically as a line. Differences were not statistically significant

Mean ECD did not change significantly from baseline to any postoperative follow-up time point (*p* > 0.05). At baseline, mean (± SD) ECD was 2240.8 ± 363.4 cells/mm^2^. ECD was 2175.6 ± 443.7 cells/mm^2^ (*p* > 0.05) and 2336.5 ± 388.6 cells/mm^2^ after 12 and 24 months, respectively. The mean ECD at all time points is shown in Fig. 3.

### Success rates

The Kaplan–Meier analysis showed a complete success rate of 58.1% with a target IOP level of ≤ 15 mmHg after 12 months and 52.9% after 24 months. Qualified success with a target IOP level of ≤ 15 mmHg was achieved in 59.7% of eyes after 12 months and 54.6% after 24 months.

**Fig. 4:**
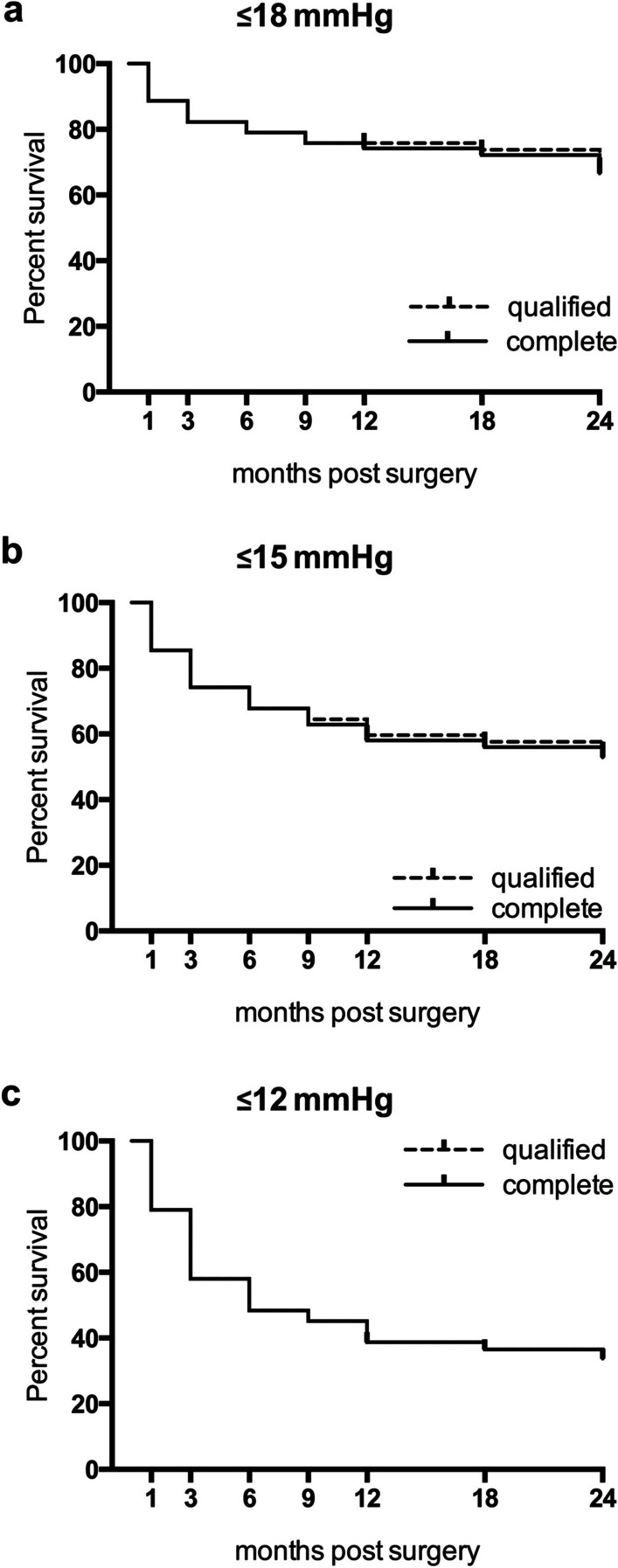
Kaplan–Meier survival plot illustrating the probability of success after PMS implantation in the entire study population. The probabilities of “complete success” and “qualified success” with a target IOP ≤ 18 mmHg (a), ≤ 15 mmHg (b), and ≤ 12 mmHg (c) are shown

The Kaplan–Meier curves for each target IOP level are shown in Fig. 4.

In the additional analysis of the two subgroups "previous surgery" (*n* = 13) and "no previous surgery" (*n* = 49), the success rates tended to be higher for the "no previous surgery" group but without statistical significance (*p* > 0.05). In the "no previous surgery" group, 61.2% and 55.1% achieved complete success with IOP ≤ 15 mmHg at 12 and 24 months. Qualified success was achieved in 63.3% and 57.3% at 12 and 24 months. In contrast, in the "previous surgery" group, complete and qualified success with IOP ≤ 15 mmHg was achieved in 46.2% at both 12 and 24 months (*p* > 0.05).

**Fig. 5:**
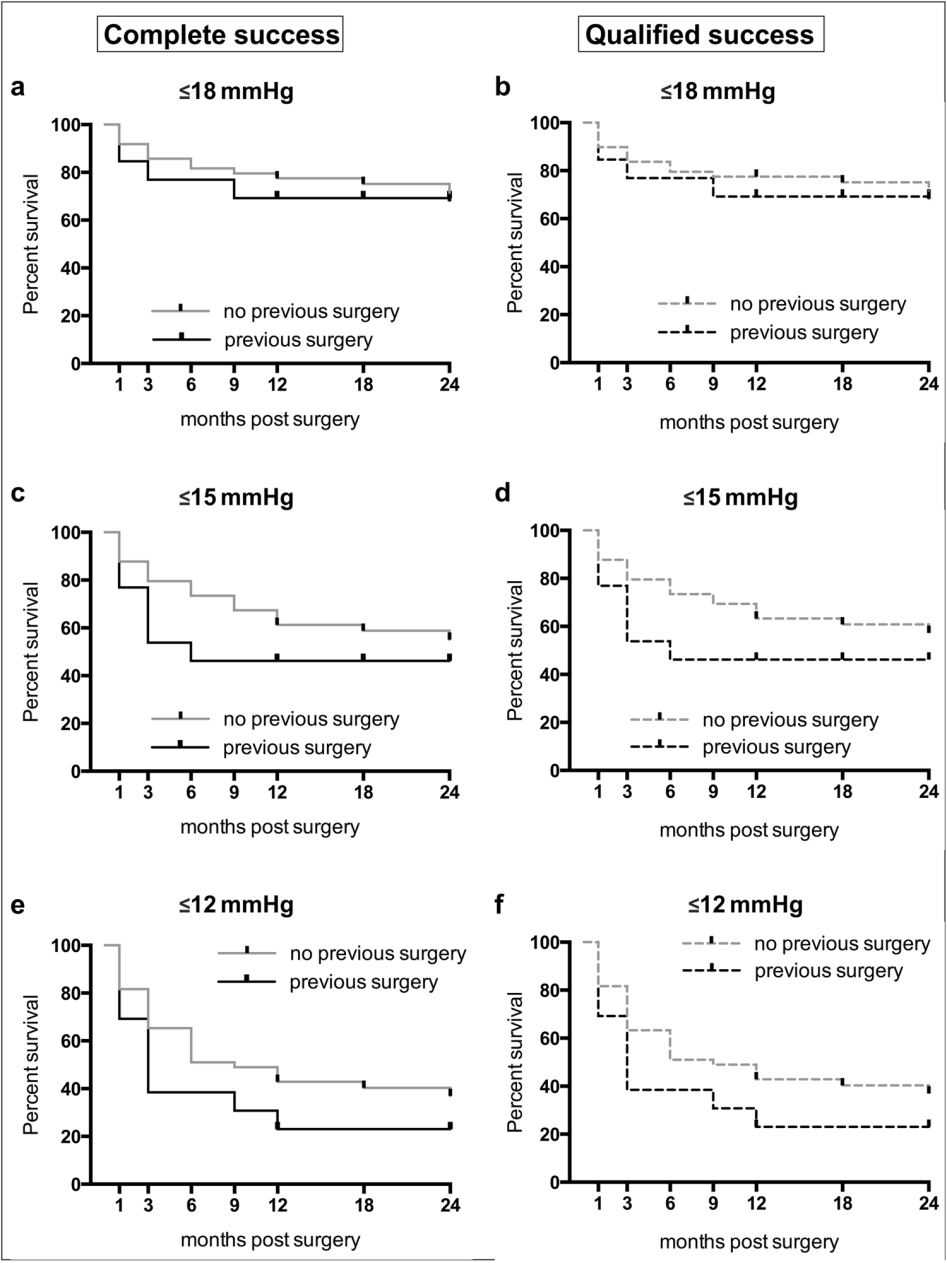
Kaplan–Meier survival plots illustrating the probability of success after PMS implantation for eyes with “previous surgery” and eyes with “no previous surgery”. a, c and e display the probabilities of “complete success” which was defined as an IOP reduction of ≥ 20% and achievement of the respective target IOP without IOP-lowering medication. b, d and f display the probabilities of “qualified success” with the respective target IOP. The survival probabilities with a target IOP ≤ 18 mmHg, ≤ 15 mmHg and ≤ 12 mmHg are shown. Differences were not statistically significant (*p* > 0.05)

The Kaplan–Meier curves for the two subgroups are shown in Fig. 5.

### Needling and incisional bleb revision

A needling procedure was performed at least once in 24 (38.7%) of the studied eyes. An incisional bleb revision was necessary in 16 (25.8%) of the eyes.

A primary incisional bleb revision without previous needling was performed in seven eyes (11.3% of all studied eyes). Of the 24 patients who had at least one needling, nine (14.5% of all studied eyes, 37.5% of eyes with needling) received an incisional bleb revision during the follow-up period. The average time between first needling and subsequent incisional bleb revision was 3.2 ± 3.0 months. In contrast, three eyes first received an incisional bleb revision and then a needling during the postoperative period, with an intervening interval of 2.0 ± 1.3 months. Thirty-four eyes (54.8%) did not require any bleb intervention after PMS implantation. The mean (± SD) numbers of needlings and incisional bleb revisions performed per eye were 0.6 ± 0.9 and 0.3 ± 0.6, respectively. The mean time to the first needling was 9.1 ± 7.0 months, and the mean time to first incisional bleb revision was 10.7 ± 6.8 months.

In the group of eyes with "previous surgery", the mean (± SD) numbers of needlings and incisional bleb revisions performed per eye were and 0.8 ± 0.9 and 0.4 ± 0.9, respectively, while in the "no previous surgery" group the mean (± SD) numbers were 0.5 ± 0.9 and 0.3 ± 0.6, respectively.

### Complications

Six eyes (9.7%) showed a hypotony with choroidal detachment and a flat anterior chamber within the first 4 postoperative weeks. In five of those, the anterior chamber was filled with a high viscosity viscoelastic substance after a mean of 14.8 ± 8.2 days postoperatively. In three of the eyes, this was repeated after a mean of 22 ± 7.2 days after PMS implantation. This approach resulted in complete recovery of choroidal detachment and the flat anterior chamber in all eyes. One eye recovered spontaneously after 14 days. We did not observe any cases of persistent hypotony. No sight-threatening complications were observed throughout the follow-up period. None of the eyes required additional glaucoma surgery or laser treatment.

## Discussion

The results of this study showed that PMS implantation achieved substantial IOP reductions of 48.5% and 54.8% after 12 and 24 months, respectively. Our study demonstrated that two thirds of the eyes retained an IOP level of ≤ 18 mmHg without any IOP-lowering medication after 24 months. These findings are consistent with those of previous studies demonstrating an effective decrease in IOP and the number of IOP-lowering medication after PMS implantation [7–9]. Fea et al. [8] observed complete and qualified success, with an IOP reduction of ≥ 20% and a target IOP ≤ 18 mmHg, in 26.0% and 58.7% of cases after 12 and 24 months, respectively, in a retrospective multicentre study of primary open-angle and pseudoexfoliative glaucoma. With a target IOP of ≤ 18 mmHg, our data show higher complete and qualified success rates of 74.2% and 75.8%, respectively, after 12 months. Compared to our results, Ibarz Barberá et al. [7] found a slightly lower mean IOP reduction of 38.7% at the final follow-up time point, with a mean follow-up period of 11 months. The authors defined complete and qualified success as reaching an IOP level of 6–17 mmHg and a minimum IOP reduction of 20% without or with medication, respectively, and observed complete and qualified success in 70.3% and 12.5% of patients, respectively, at the final visit [7]. Although the data are not directly comparable due to different success criteria and follow-up periods, the complete success rate appears to be similar to that in our study, as we observed a 74.2% complete success rate of IOP ≤ 18 mmHg after 12 months.

Interestingly, our sub-analysis showed that eyes with previous surgery tended to have lower success rates than those without previous surgery, even though the difference was not statistically significant. This may be due to pre-existing conjunctival scarring in the pre-operated eyes. It is also conceivable that these are patients who generally have a greater tendency to scar and have therefore already had failed glaucoma surgery in the past. The same reasons may also be responsible for the comparatively higher needling rate in eyes with previous surgery. The impact of previous surgery on the success rate of PMS deserves further investigation in a larger cohort.

Further aims of this study were to assess the safety and, specifically, the potential impact of PMS implantation on CECs. The complications observed in this study were manageable and had no long-term sequelae. Notably, ECD did not change significantly after 24 months.

The potential decline of ECD is one of the major concerns regarding the safety of glaucoma implants and drainage devices. Knowledge of the specific risks of the devices is crucial for the selection of the most favourable surgical procedure and for adequate patient counselling and education. It is worth noting that previous studies have reported concerns about endothelial cell loss (ECL) associated with other drainage devices, such as the CyPass Micro-Stent and the Ahmed glaucoma valve. In 2019, significant ECL was demonstrated up to 5 years after implantation of the CyPass Micro-Stent [3, 10]. This led to the withdrawal of the device from the market [3]. Significant decreases of CECs by 15.3% at 12 months and 18.6% at 24 months were also reported for the Ahmed glaucoma valve [11]. Similarly, the Ahmed glaucoma valve was independently associated with a significant ECL in children with uveitic glaucoma [12].

Ibarz Barberá et al. [13] reported that PMS implantation was associated with an ECL of 7.4% 12 months after surgery. ECL began immediately after surgery and continued over time, albeit at a slower rate after the first year. Position of the PMS, anterior chamber depth, hypotony and the presence of peripheral anterior synechia were factors influencing ECD changes. This study demonstrated the importance of proper positioning of the PMS. The proximity of the PMS to the corneal endothelium affected the ECL, with PMS implants located farther from the endothelium showing lower rates of ECL [13]. Similarly, the proximity of the tube was a major risk factor for ECL in patients with a Baerveldt drainage device or an Ahmed glaucoma valve [14, 15]. Baker et al. [16] reported mean rates of ECL of 5.2% and 6.9% 1 year after PMS implantation or trabeculectomy, respectively. Chamard et al. [5] reported significant ECL 5 years after PMS implantation in two cases. In one of these cases, anterior segment OCT and ultrasound biomicroscopy indicated a probable backward movement and a short intracameral portion of the device causing a corneal touch. In the second case, the authors assumed an inflammatory reaction. This case series indicates that ECL might be a slowly progressive process that takes years to become evident.

In contrast, we did not observe any significant ECL over the 24-month follow-up period of our study, which supports the intermediate-term safety profile of the PMS. A possible explanation for this discrepancy to the reported ECL in other studies is that at the time of the start of this study the learning curve of the surgeon was already completed, so that the risk of an ECL due to improper placement of the stent too close to the endothelium was minimized. On the other hand, no anterior segment OCT was performed in this study, so that no precise statement can be made about possible differences in the placement of the PMS in the anterior chamber. Further studies with anterior segment OCT and long-term observation and would be of great interest.

Similarly, Qidwai et al. [17] found no significant ECL over a 24-month study period; however, endothelial cell counts were available for only 12 of 48 patients. Furthermore, studies by Jamke et al. [18] and by Steindor et al. [19] found no significant ECL 12 months after PMS implantation. Notably, however, the current study is the only one investigating the impact of the PMS on the corneal endothelium with a follow-up period of at least 2 years.

Regarding pre- and postoperative complications, we did not observe any cases of permanent hypotony in our study. Filtering stent-based procedures are reported to have lower rates of long-term hypotony than trabeculectomy. A retrospective cohort study by Van Lancker et al. [20] reported no cases of persistent hypotony after PMS, in contrast to trabeculectomy. Chronic hypotony was the main reason for failure in the trabeculectomy patients in this study [20]. Several other studies showed a higher rate of prolonged hypotony with trabeculectomy than with PMS [21]. This is explained by the flow-limiting design of the PMS based on the Hagen–Poiseuille equation, which is intended to prevent permanent hypotony [22].

We observed that BCVA remained stable, without a significant reduction over the study period. This finding is reassuring, as it indicates that PMS implantation does not compromise visual acuity, which is crucial for maintaining the quality of life of glaucoma patients. Several studies found no significant alterations in BCVA after PMS implantation [23, 24]. In contrast, a cross-sectional, multicentre, retrospective study found a visual loss of > 2 Snellen lines in 5.6% of 428 studied eyes after trabeculectomy [25]. Another study reported 8.0% permanent vision loss after trabeculectomy in a large retrospective study [26].

We acknowledge that the single-centre design and some missing follow-up visits might introduce bias despite meticulous planning of this prospective study. In addition, the follow-up period of 24 months might have been too short to detect a significant rate of ECL. Consequently, we plan to prolong the study to gain a more comprehensive understanding of the safety and efficacy of the PMS.

## Conclusion

Our study provides valuable insights into the safety and efficacy of the PMS in the treatment of medically uncontrolled open-angle glaucoma. Notably, ECD did not change significantly after 24 months. We found that 66.6% of eyes had an IOP of ≤ 18 mmHg and were free from IOP-lowering medication after 24 months. The complications were manageable or self-limiting and had no long-term sequelae.

## Declarations

The study adhered to the principles of the Declaration of Helsinki of 1964. Prior to surgery, all patients provided informed consent, along with additional written consent for study participation. Ethical approval was granted by the local institutional ethics committee of the University of Tübingen (project number: 037/2021BO2).
